# Effect of perioperative fluid balance on the postoperative outcomes of patient after esophagectomy

**DOI:** 10.6026/973206300211932

**Published:** 2025-07-31

**Authors:** Sofia Jaswal, Harsimran Singh Walia, Atin Goyal, Lalita Gouri Mitra, Nimish Singh, Vikram Singh

**Affiliations:** 1 Department of Anaesthesia, Critical Care and Pain, Homi Bhabha Cancer Hospital and Research Centre, Punjab, India; 2 Department of Surgical Oncology, Homi Bhabha Cancer Hospital and Research Centre, Punjab, India

**Keywords:** Esophagecomy, fluid balance, perioperative, postoperative outcomes

## Abstract

The effect of perioperative fluid balance on the postoperative complications and outcomes among patients who underwent esophagectomy surgery at a rural tertiary health care centre over 1 year is of interest. The effect of intraoperative and postoperative fluid balance on the postoperative complications and length of ICU and hospital stay was studied. Postoperative day 1 (POD1) balance (p-value 0.05) and cumulative fluid balance (p-value 0.017) were significantly associated with postoperative ICU stay. As the POD1 balance was significantly associated with the ICU stay, we divided the patients into low- and high-balance groups based on the mean POD1 balance (1484.74 mL), and ICU stay was found to be significantly longer in the high-balance group (p-value 0.023). POD1 balance and cumulative fluid balance appear to be the most influential variables, exhibiting a substantial positive relationship with ICU stay.

## Background:

Perioperative fluid balance is thought to be crucial for the treatment of patients with esophageal cancer who undergo surgery [1]. The incidence of postoperative complications after esophagectomy is high (65%), which includes pneumonia in 29% of patients and anastomotic leak in 19% of patients [2]. Postoperative complications after esophagectomy may impact prognosis. They lower the esophageal patients' overall survival rate [3]. To prevent postoperative complications, perioperative management should be carefully considered [4]. Various studies have shown that adverse surgical outcomes and postoperative complications are affected by intraoperative and postoperative fluid overload in patients of esophageal cancer surgery, but the evidence is still inconclusive [5, 6]. Therefore, it is of interest to effect of perioperative fluid balance on postoperative complications and outcomes in patients with esophageal cancer after surgery.

## Materials and Methods:

This was a single-centre, retrospective study in which all the patients with esophageal cancer who underwent video-assisted thoracoscopic (VATS) esophagectomy at a rural tertiary health care centre between 1st July 2022 and 30th September 2023 were included. This study aimed to investigate how perioperative fluid balance affected postoperative complications and the average duration of hospital and ICU stays. The intraoperative records were checked from the electronic medical records, intraoperative anaesthesia charts, and postoperative records were recorded from the intensive care unit (ICU) charts. Perioperative fluid balance was calculated by subtracting the fluid eliminated from the body through all means from the fluid given during the intraoperative and postoperative period till day 2 (POD 2) during the ICU stay. Intraoperative, POD0, POD1, POD2, and cumulative fluid balance were noted. Clavien-Dindo classification grade ≥ 2 was used to describe the postoperative complications [7]. Pneumonia was characterized as new pulmonary infiltrates in chest X-ray with clinical signs and symptoms of infection, such as purulent sputum, reduced oxygenation, and new-onset fever [8]. Arrhythmia in our study included atrial fibrillation (AF), which is one of the most common arrhythmias in postoperative esophagectomy patients [9]. Clinical evaluation and CT scan results showed anastomotic leakage. Symptoms of hoarseness led to the diagnosis of recurrent nerve palsy that was later verified by bronchoscopy [10]. Chest drain output's milky color, amount, or quality, or pleural fluid triglycerides more than 110 mg/dL, were used to diagnose chylothorax [11]. Reduced oxygenation, bedside echocardiography, and computed tomography pulmonary angiography were used to diagnose pulmonary embolism [12]. Purulent discharge from the surgical site with positive cultures was referred to as a surgical site infection (SSI). Furthermore, adopting the Kidney Disease Improving Global Outcomes (KDIGO) clinical practice recommendations, acute kidney injury (AKI) was defined as decreased urine output and elevated serum creatinine (Cr) [13]. The average duration of stay in the intensive care unit and hospital was noted. Any patient requiring reintubation or readmission was noted.

## Statistical analysis:

Descriptive analysis was used in the compilation of the data. Chi-square test or Fisher's exact test was used to find the association of categorical variables. Graphical methods and various statistical tests were used to assess the normality of the data. To ascertain the relationship between the variables, both logistic regression and linear regression analysis were applied. The p-value was considered to be significant at 0.05. All the statistical analysis was performed using IBM SPSS (Statistical Packages for Social Sciences, version 28.0. Armonk, NY: IBM Corp).

## Results:

43 patients underwent video-assisted thoracoscopic (VATS) esophagectomy at our centre. Of these, 8 patients' intraoperative records were not found, 5 patients' postoperative ICU charts couldn't be traced, and 3 patients were lost to follow-up. So, we recruited 27 patients in this study who underwent VATS esophagectomy in the last one year. The mean age of the participants was 55.93 years, the mean weight was 63.26 kg, and the average height was 161.41 cm. 66.7% of patients were female and 33.3% of patients were male. The mean haemoglobin (Hb) was 12.23 g/dL and the mean albumin was 3.76 g/dL preoperatively. Of these patients, 55.6% were assigned to ASA class 2 and 33.3% to ASA class 1. Table 1 (see PDF) shows the input-output and fluid balance for intraoperative and postoperative days 0-1. Most common postoperative complications were anastomotic leak (29%), AKI (25%), arrhythmia (22%), along with other complications as are listed in Table 2 (see PDF). The average hospital stay was 13.33 days, and the average ICU stay was 6.9 days (Table 1 - see PDF). There was an 18.5% incidence of reintubations, and 11.1% of patients required readmission (Table 2 - see PDF). In our study, postoperative day 1 (POD1) balance was a significant independent predictor (p value = 0.05) of postoperative ICU stay. Specifically, each unit increase in POD1 balance increases the ICU stay by 0.003 days. Moreover, cumulative fluid balance was also associated with increased postoperative ICU stay, and the difference was statistically significant (p-value 0.017). This implies that a higher fluid balance on postoperative day 1 and cumulative fluid balance are associated with a longer stay in the ICU or difficulties during recovery. However, intraoperative and POD2 balance were not significant predictors of ICU stay in our study, indicating that they have no substantial or statistically significant impact on ICU stay in this dataset. Hospital stay was not found to be significantly associated with the intraoperative, POD1, POD2, and cumulative fluid balance. However, POD1 balance (p = 0.074) was close to significance, indicating a possible influence on hospital stay duration (Table 3 - see PDF). As the POD1 balance was significantly associated with the ICU stay, we divided the patients into low and high balance groups based on the mean POD1 balance (1484.74ml). Patients in the low balance group received less than 1484.74 ml of fluid on POD1, and patients in the high balance group received more than 1484.74 ml of fluid on POD1. The postoperative complications were compared between both groups, and the difference was not statistically significant. ICU stay in the high balance group was more (9.23 days) than the low balance group (4.86 days), and this difference was statistically significant (p-value 0.023). However, the hospital stay was not significantly different between the two groups (Table 4 - see PDF).

## Discussion:

This study found that high fluid balance on POD1, as well as high cumulative fluid balance, is associated with an increase in the ICU stay. This implies that each unit increase in POD1 balance increases the ICU stay by 0.003 days. Intraoperative fluid balance, fluid balance on the day of surgery, and POD 2 fluid balance were not found to be associated with the ICU stay. The possible reason for this finding could be that the high fluid balance may increase the extravascular fluid in the lung, which can lead to increased oxygen requirements. Additionally, fluid overload from increased fluid balance might cause edema and postpone recovery. High cumulative fluid balance, especially on POD1, can lead to increased ICU stay, as it may delay the return of the normal physiological reserves after esophagectomy because of the excessive extravascular fluids and edema. However, the total hospital stay was not found to be associated with fluid balance on any day, as well as the cumulative fluid balance in this study. Esophagectomy is amongst the most invasive and high-risk gastrointestinal cancer surgeries [2]. The postoperative complication rate is high in these surgeries [3]. Various studies have demonstrated a positive relation between the positive perioperative balance and the postoperative complications like pneumonia, anastomotic leak, and AKI [4, 5 and 6]. The effect of perioperative fluid balance on postoperative complications was evaluated by Kubo and colleagues after minimally invasive esophageal cancer surgery. They reported that anastomotic leakage as well as acute pneumonia within 7 postoperative days were more common in patients with a high fluid balance (>3,000 mL) on POD 1 [14]. Additionally, Hikasa *et al.* investigated the relationship between postoperative fluid balance and esophageal resection patients [15]. The incidence of surgical complications was evaluated among patients with fluid balances of more than or less than 4311 ml. They found that fluid overload had a negative association with postoperative complications. They reported that the incidence of postoperative complications, including arrhythmia, deep venous thrombosis, other thromboses, and pneumonia, was significantly higher in patients who received a high perioperative fluid balance [15].

However, we didn't find any significant relation with the perioperative fluid balance and the postoperative complications like pneumonia, anastomotic leak, arrhythmias, and others. A previous study by Myles *et al.* had demonstrated a higher incidence of postoperative AKI after major abdominal surgery in a restrictive fluid protocol as compared to the liberal fluid protocol [16]. However, we didn't observe any association between the infusion fluid volume and the incidence of AKI. A previous study by Takahashi *et al.* also demonstrated similar results, with no association observed between the perioperative volume of fluid administered and the incidence of postoperative AKI in 300 patients enrolled for minimally invasive esophagectomy [17]. There are several limitations to our study. First of all, this study was conducted in a single institution and was retrospective in nature. To confirm the results of this study, large-scale prospective studies need to be conducted at various centres. A smaller sample size was another limitation of the study. Moreover, the anaesthetists managing the patients during surgery and ICU were random. Anaesthesia was managed based on the judgment of the attending anaesthetist since there are no established protocols for fluid management. Hence, the fluid administered may differ between different anaesthetists. To clarify the relationship between perioperative fluid management and postoperative outcomes in patients undergoing esophagectomy, more prospective studies with well-defined protocols for perioperative hemodynamic and fluid management and sizable sample sizes are necessary.

## Conclusion:

POD1 balance and cumulative fluid balance appear to be the most influential variables, exhibiting a substantial positive relationship with ICU stay. However, intraoperative or POD2 fluid balance measurements have no significant effect on ICU stay duration. Nonetheless, cumulative fluid balance does not have a significant impact on the postoperative complications and the length of hospital stay. Hence, we suggest that fluid balance in postoperative patients' needs to be controlled after esophagectomy, especially at POD 1.
